# Age at first sex and other determinants of multiple sexual partnerships among sexually active adolescent girls in Ghana: a regression analysis of the 2022 Ghana demographic and health survey

**DOI:** 10.1186/s41182-024-00644-x

**Published:** 2024-10-22

**Authors:** Desmond Klu, Micheal Larbi Odame, Evelyn Acquah, Charity Akpene Dansu

**Affiliations:** 1 Institute of Health Research, University of Health and Allied Sciences, Ho, Ghana; 2 Department of Sustainable Development and Policy, University of Environment and Sustainable Development, Somanya, Ghana; 3 Ghana Education Service, Kotobabi TMA School, Tema West, Ghana

**Keywords:** Age at first sex, Multiple sexual partnership, Adolescent girls, Survey, Ghana

## Abstract

**Background:**

Early sexual debut is a widely recognized public health issue due to its influence on lifetime multiple sexual partners which can lead to complications such as teenage pregnancy, abortion, HIV/AIDS and other sexually transmitted infections. However, there is paucity of research evidence on sexual behaviour such as age at first sexual intercourse and the number of sexual partnerships among adolescent girls in Ghana. This study aims to examine the influence of age at first sexual intercourse and other determinants on multiple sexual partnerships among adolescent girls aged 15–19 years in Ghana.

**Methods:**

Data for this study were obtained from the 2022 Ghana Demographic and Health Survey (GDHS) conducted between October 17, 2022 and January 14, 2023. A total weighted sample of 719 sexually active adolescent girls who reported having sexual partners was extracted from the women’s data file. Data were analyzed with SPSS version 27, employing multilevel logistics regression modelling. Statistical significance was set at p < 0.05.

**Results:**

The average age at sexual debut for adolescent girls aged 15–19 years in Ghana is 15.5 years. A little over half (51.6%) of adolescent girls reported having two or more lifetime sexual partners. Adolescent girls who initiated sex before reaching age 15 were more likely (aOR = 2.00; C.I 1.39–2.87) to have multiple partners compared to those who had their first sex before attaining age 20. After controlling for other factors, girls who had their sexual debut before age 15 had higher odds (aOR = 1.85; C.I:1.30–3.31) of engaging in multiple sexual partnerships. Girls living female headed households (aOR = 2.25; C.I:1.18–4.29] and consuming alcohol (aOR = 2.38; C.I 2.38–1.09–5.17) had higher odds of having multiple sexual partners.

**Conclusion:**

The study findings show that early sexual debut, living in female headed household, and consuming alcohol are strong predictors of multiple sexual partnerships among adolescent girls in Ghana. It suggests the need for policies promoting delayed sexual debut and empowering adolescents to make informed decisions to improve sexual health outcomes.

## Background

The stage of adolescence represents a critical transition from childhood to adulthood, characterised by changes in psychosocial, cognitive, physiological and behavioural aspects [1, 2]. One major change during this transition is the emergence of adolescent sexuality, often evidenced by curiosity and anxiety towards sexual decision-making processes and initial experiences [3]. These explorations expose adolescents to sexual vulnerabilities and negative sexual health outcomes such as abortions, sexually transmitted infections (STIs), including Human Immunodeficiency Virus (HIV)/Acquired Immune-Deficiency Syndrome (AIDS), sexual abuse and unplanned pregnancies [4].

According to the latest World Health Organisation (WHO) report on adolescent health, adolescents aged 10–19 years constitute 1.2 billion, accounting for 18% of the world’s population [5]. Notably, approximately 90% of these adolescents reside in lower and middle-income countries [6]. Globally, by age 19, approximately 70% of adolescent boys and girls are reported to be sexually active [7], while the proportion of adolescents engaged in multiple sexual partnerships has significantly reduced worldwide [8]. In sub-Saharan Africa (SSA), there are an estimated 250 million adolescents (10–19-year-olds), a number expected to increase to 24% by 2030 [9]. A recent study by Federe and colleagues [10] found that the prevalence of early sexual debut among adolescent girls in SSA is estimated at an average of 46.4%, with the lowest of 38.9% in East Africa and 58.4% in the Central African region.

In Ghana, according to the 2021 Population and Housing Census (PHC) report, the population of adolescent girls (aged 10–19 years) increased from 2,737,392 in 2010 to 3, 316,773 in 2021 [11]. The 2022 Ghana Demographic and Health Survey further reports that 317 adolescents (15–19 years) sampled during the survey are sexually active. Recent studies in Ghana in 2022 have reported that the average age for first sexual contact among adolescent girls was 15.1 years, with 36% reporting having multiple sexual partners [11–13]. Additionally, 26.7% had their first sexual intercourse before attaining the age of 16 [12], while other studies found that 55% of adolescent girls had their first sexual debut before reaching the age of 19 [13].

Age at first sexual debut is a strong determinant of other risky sexual behaviours such as multiple sexual partnerships [14–17], unprotected sex and negative sexual outcomes such as HIV/AIDS [18–20]. Earlier studies have reported that adolescents who experience their first sexual intercourse at an early age are more at risk of poor and adverse sexual and reproductive health outcomes [4, 14, 16, 21, 22]. Furthermore, early sexual debut has been highly associated with decreased contraceptive use, sexual autonomy, rights and abstinence [23–26], which can negatively affect the sexual and reproductive health and quality of life among adolescent girls and young women. This phenomenon of increasing risky sexual behaviour is prevalent not only in developed countries but also in developing and less developed regions such as sub-Saharan Africa (SSA), with high rates of HIV/AIDS and teenage pregnancy.

Our study relayed on the Ecodevelopmental Theory propounded by Szapocznik and Coatsworth [27], this theory provides a framework of sexual activity in relation to other demographic, social and risk factors. Risky sexual acts are viewed within a social context that includes multiple factors interacting at different levels including, intrapersonal, interpersonal, household, community, social and national levels. These different levels of interactions fundamentally influence sexual behaviours of adolescents. Consistent with Ecodevelopmental Theory, this study linked risk sexual behaviour (multiple sexual partnerships) to demographic, household, social and other risk factors.

Several studies using nationally representative surveys [13–15, 28–33], school-based cross-sectional surveys [34–36] and community-based surveys [37, 38] have examined age at first sex, demographic, household, social and risk factors predicting multiple sexual partnerships among adolescents and young people.

Although evidence from the literature reveals that adolescents who initiate sex at an earlier age are more likely to have multiple sexual partners, the combined effect of other mediating factors such as individual, household and risk factors influencing the age at first sex on multiple sexual partnerships among adolescent girls aged 15–19 years is understudied to the best of our knowledge. This study is particularly important considering the increasing rate of teenage pregnancy among adolescent girls in Ghana from 13% in 2008 to 15% in 2022 [39, 40]. Therefore, it is critical to study the drivers of these negative sexual and reproductive health outcomes among females. This study focuses on adolescent girls for the following reasons: in Ghana and some African countries, females engage in their first sexual intercourse at an earlier age compared to boys, where 8.2% of young women and 3.6% of young men had their first sexual intercourse before reaching age 15 [41–43], and a higher risk of HIV infection is found among young women in SSA. These estimates threaten Ghana’s efforts to achieve the Sustainable Development Goal (SDG) 3, which aims at ensuring healthy lives and promoting well-being for all ages. The study, therefore, examines the influence of age at first sex and other individual, household and risk factors on multiple sexual partnerships among adolescent girls aged 15–19 years in Ghana using evidence from the 2022 Ghana Demographic and Health Survey.

## Method

### Study design and population

The study utilised data from the 2022 Ghana Demographic and Health Survey (GDHS), a nationally representative cross-sectional survey. The GDHS collects information on various topics including housing characteristics, household population, marriage and sexuality, fertility and fertility preferences, family planning, infant and child mortality, maternal health, and child and early development, nutrition of children and women, malaria, HIV and AIDS related knowledge, attitudes, and behaviours, HIV prevalence, adult health and lifestyle, women’s empowerment and demographic and health outcomes. This study specifically focuses on sexually active adolescent girls aged 15–19 who have sexual partners.

### Sampling procedure and sample size

The sampling frame used for the 2022 GDHS was the updated frame prepared by the Ghana Statistical Service (GSS) based on the 2021 Population and Housing Census (PHC). The sampling procedure employed in the 2022 GDHS was a stratified two-stage cluster sampling method, designed to yield representative results at the national level, for both urban and rural areas, and across each of the 16 regions for most DHS indicators. In the first stage, 618 target clusters were selected from the sampling frame using probability proportional to size (PPS) for urban and rural areas within each region. The target number of clusters was then selected with equal probability through systematic random sampling of the clusters identified in the first phase, for both urban and rural areas in each region. In the second stage, after selecting the clusters, a household listing and mapping operation was conducted in all the selected clusters to develop a comprehensive list of all households within each cluster. This list served as the sampling frame for selecting the household sample. The household listing was conducted using tablet computers with software provided by The DHS program. A fixed number of 30 households in each cluster was randomly selected from the list for interviews.

In the 2022 GDHS, sexually active adolescent girls aged 15–19 who reported having sexual partners, whether single or multiple, were extracted from the larger dataset. The women’s data file used in this study was weighted, resulting in a sample of 2,682 adolescent girls aged 15–19 years. From this sample, adolescent girls who reported having sexual partners in their lifetime were filtered out, resulting in a total weighted sample of 719.

## Measurement of study variables

### Dependent variable

The variable of interest in this study was sexual partnership among adolescent girls aged 15–19 years. Sexual partnership was defined as either having a single sexual partner or multiple (2 or more) sexual partners in their lifetime. The following question was used to measure sexual partnerships: “In total, with how many different people have you had sexual intercourse in your lifetime?”. Adolescent girls who had a single sexual partner in their lifetime were coded as “0”, while those who reported having two or more sexual partners were coded as “1”.

### Predictor variables

#### Main predictor variable

The main predictor variable in this study was age at first sex. This variable was categorised into two groups: (i) adolescent girls who had sex before reaching the age of 15 years (those who experienced sexual debut between 1 and 14 years) and (ii) adolescent girls who had sex before reaching the age of 20 years (those who had their first sexual intercourse between 15 and 19 years).

### Other predictor variables

#### Individual socio-demographic factors

The individual socio-demographic factors in the study comprised educational level (no education, primary, secondary +), religion (Orthodox, Pentecostal/Charismatic, Other Christian, Islam, No religion) and ethnicity (Akan, Ga/Dangme, Ewe, Mole-Dagbani, Gurma).

### Household level factors

Household factors considered for this study included sex of household head (male, female), age of household head (20–29, 30–39, 40–49, 50–59, 60–69, 70 +) and household wealth quintile (poorest, poorer, middle, richer, richest).

### Community level factors

In this study, we considered the following as community level factors: ecological zone of residence (Northern zone, Middle zone and Coastal zone), place of residence (urban and rural).

### Social and risk level factors

The study also considered other social and risk factors such as alcohol consumption in the last 4 weeks (did not consume alcohol, consumed alcohol, never consumed alcohol), contraceptive use by method type (use no method, use traditional method, use modern method), cigarette smoking (did not smoke, smoke), ever tested for HIV (no, yes), had any STI (no, yes), ever heard of STI (no, yes) and ever heard of AIDS (no, yes). The choice of these social and risk factors was informed by previous studies [4, 17, 19–21].

### Analytical technique

The data were analysed using SPSS version 27. Three stages were followed in the data analysis technique. In the first stage, we employed simple descriptive statistics to provide an overview of the dependent and predictor variables. The second stage involved a cross-tabulation of adolescent girls’ age at first sex and sexual partnership, as well as all individual socio-demographic, household, social and risk factors against multiple sexual partnerships among adolescent girls aged 15–19 years. We use chi-square analysis for this purpose and statistical significance was set at a p-value of 0.05.

In the third stage, six models were developed using multilevel binary logistic regression modelling to examine the effect of age at first sex, along with individual, household, community and social and risk factors on multiple sexual partnerships among adolescent girls aged 15–19 years in Ghana. This analytical technique was used by earlier studies [44, 45]. The first model examined the relationship between age at first sex alone and multiple sexual partnership. Individual level factors were included in the second model along with age at first sex, while household level factors and age at first sex were incorporated in model III. Model IV regressed age at first sex and community level factors on multiple sexual partnership, while model V examined the effect of age at first sex and social and risk level factors on multiple sexual partnership. The final model included age at first sex together with individual, household, community, social and risk levels factors. Each model provided an adjusted odds ratio (aOR) and their corresponding 95% confidence intervals (CIs) to assess the relationships.

## Result

### Background characteristics of sexually active adolescent girls 15–19 years in Ghana

Table 1 presents the individual and household characteristics of sexually active adolescent girls aged 15–19 who have sexual partners in Ghana. The results show that the highest proportion of adolescent girls have attained secondary or higher education level (81.6%), while approximately 48% reside in the middle belt zone of the country. A little over half of adolescent girls reside in rural areas (50.6%), with approximately 45% belonging to the Pentecostal or Charismatic faith. The majority of adolescent girls aged 15–19 years in Ghana belong to the Akan ethnic group (51.8%).

**Table 1:** Individual and household characteristics of sexually active adolescent girls in Ghana

Individual and household factors	Weighted Sample n = 719	%
Education Level		
No education	21	2.9
Primary	112	15.5
Secondary +	586	81.6
Religion		
Orthodox	161	22.4
Pentecostal/charismatic	326	45.3
Other christian	110	15.3
Islam	100	13.9
No religion	23	3.1
Ethnicity		
Akan	372	51.8
Ga/dangme	52	7.2
Ewe	103	14.3
Mole-Dagbani	96	13.4
Gurma	49	6.9
Other	46	6.4
Sex of household head		
Male	357	49.7
Female	362	50.3
Age of household head		
20–29	76	10.6
30–39	85	11.8
40–49	199	27.7
50–59	179	24.9
60–69	124	17.2
70 +	56	7.8
Household wealth quintile		
Poorest	132	18.3
Poorer	182	25.3
Middle	**205**	28.4
Richer	128	17.8
Richest	73	10.1

Source: computed from 2022 Ghana demographic and health survey (GDHS)

Regarding their household characteristics, most adolescent girls belong to households headed by females (50.3%), with a higher proportion of household heads within the age bracket 50–59 (24.9%) while most of them belong to the middle household wealth quintile (28.4%).

Table 2 illustrates the social characteristics and risk factors associated with adolescent girls aged 15–19 with sexual partners. Nearly half (49.5%) of adolescent girls with sexual partners had not used any method of contraception, with approximately 18% having consumed alcohol in the past 4 weeks preceding the survey. Less than one percent (0.8%) indicated they had smoked cigarettes and a higher proportion had never tested for HIV (76.9%). The majority of these adolescent girls have ever heard of STIs (96.2%) and AIDS (94.8%) while only 8.3% indicated having STIs.

**Table 2:** Community and social and risk factors among adolescent girls (15–19) years in Ghana

Community level factors	Weighted Sample n = 719	%
Ecological zones of residence		
Northern zone	101	14.0
Middle belt	343	47.7
Coastal zone	275	38.3
Place of residence		
Urban	355	49.4
Rural	364	50.6
Social and risk factors		
Alcohol consumption in the past 4 weeks		
Did not consume alcohol in the past 4 weeks	180	25.0
Consumed alcohol in the past 4 weeks	127	17.7
Never consumed alcohol in the past 4 weeks	413	57.4
Contraceptive use by method type		
Use no method	356	49.5
Use traditional method	120	16.8
Use modern method	242	33.7
Cigarettes smoking		
Did not smoke	714	99.2
Smoke	5	0.8
Ever tested for HIV		
No	553	76.9
Yes	166	23.1
Had any STI		
No	660	91.7
Yes	59	8.3
Ever heard of STI		
No	27	3.8
Yes	692	96.2
Ever heard of AIDS		
No	37	5.2
Yes	682	94.8

Source: computed from 2022 Ghana demographic and health survey (GDHS)

### Prevalence of multiple sexual partnership and age at first sexual intercourse among adolescent girls aged 15–19 years in Ghana

**Fig. 1:**
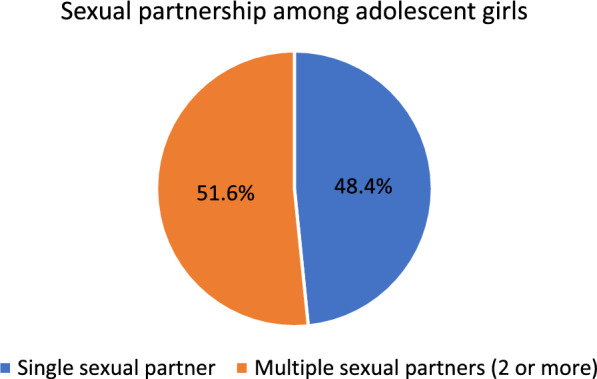
Prevalence of sexual partnership among adolescent girls. Source: computed from 2022 Ghana demographic and health survey (GDHS)

**Fig. 2:**
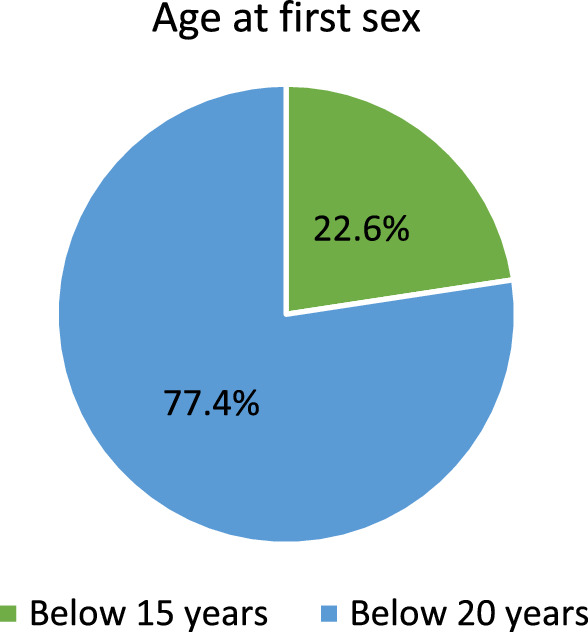
Proportion of adolescent girls’ age at first sexual intercourse

Figure 1 shows the prevalence of multiple (2 or more) sexual partnership among sexually active adolescent girls (15–19 years) in Ghana. Out of the 719 adolescent girls, 51.6% are engaged in multiple sexual partnership, while 48.4% have single sexual partner. Figure 2 illustrates the proportion of sexually active adolescent girls (15–19 years) and their age at first sexual intercourse. The result indicates that about two-thirds (77.4%) of these adolescent girls had their first sex before attaining the age of 20 years, while 22.6% had their sexual debut before reaching age of 15 years.

### Association between age at first sex, individual, household, community, social, risk factors and multiple sexual partnership among adolescent girls aged 15–19 years in Ghana

Table 3 illustrates a chi-square analysis between age at first sex, individual, household, community, social and risk factors and multiple sexual partnership among adolescent girls 15–19 years in Ghana. Age at first sexual intercourse was significantly associated with multiple sexual partnership among adolescent girls 15–19 years in Ghana at p < 0.001. The results show that a higher proportion of adolescent girls (64.8%) who had sex before reaching age 15 years reported having multiple sexual partners compared to those who had their sexual debut before reaching age 20 years (47.8%).

**Table 3:** Association between age at first sex, individual, household, community, social and risk factors and sexual partnership among adolescent girls in Ghana

Factors	Sexual partnership among adolescent girls		
	Single partners	Multiple partners (2 or more)	P-values
Age at first sex			
< 15 years	35.2	64.8	0.000***
≥ 15 to < 20 years	52.2	47.8	
Individual level factors			
Educational level			
No education	47.6	52.4	0.904
Primary	46.4	53.6	
Secondary +	48.7	51.3	
Religion			
Orthodox	55.3	44.7	0.000***
Pentecostal/charismatic	46.0	54.0	
Other christian	41.3	58.7	
Islam	59.0	41.0	
No Religion	18.2	81.8	
Ethnicity			
Akan	43.8	56.2	0.008**
Ga/Dangme	38.5	61.5	
Ewe	51.5	48.5	
Mole-Dagbani	60.4	39.6	
Gurma	63.3	36.7	
Other	50.0	50.0	
Household level Factors			
Sex of household head			0.000***
Male	55.6	44.4	
Female	41.3	58.7	
Age of household head			
20–29	43.4	56.6	0.294
30–39	46.4	53.6	
40–49	46.7	53.3	
50–59	50.3	49.7	
60–69	56.5	43.5	
70 +	40.0	60.0	
Wealth index			
Poorest	62.1	37.9	0.000***
Poorer	48.9	51.1	
Middle	40.2	59.8	
Richer	42.2	57.8	
Richest	56.2	43.8	
Community level factors			
Ecological zone of residence			
Northern zone	66.3	33.7	0.000***
Middle belt	48.7	51.3	
Coastal zone	41.1	58.9	
Place of residence			
Urban	46.0	54.0	0.213
Rural	50.7	49.3	
Social and risk factors			
Alcohol consumption			0.000***
No alcohol in the past 4 weeks	38.9	61.1	
Consumed alcohol in the past 4 weeks	38.6	61.4	
Never consumed alcohol	55.4	44.6	
Contraceptive use by method type			
Use no method	56.5	43.5	0.000***
Use traditional method	48.3	51.7	
Use modern method	36.8	63.2	
Cigarettes smoking			
Did not smoke	48.3	51.7	0.935
Smoke	50.0	50.0	
Ever tested for HIV			
No	51.9	48.1	0.000***
Yes	36.1	63.9	
Had any STI			
No	50.8	49.2	0.000***
Yes	21.7	78.3	
Ever heard of STI			
No	74.1	25.9	0.007**
Yes	47.4	52.6	
Ever heard of AIDS			
No	70.3	29.7	0.006**
Yes	47.1	52.9	

Source: computed from 2022 Ghana demographic and health survey (GDHS)

**P < 0.01; ***P < 0.001

Furthermore, a statistically significant association was found between other individual, household, community, social, risk factor and multiple sexual partnership among adolescent girls at p < 0.05. A higher proportion of adolescent girls who reside in coastal zones (58.9%) of Ghana had indicated having multiple sexual partners relative to the other zones. Again, a higher proportion of multiple sexual partnerships was found among adolescent girls with no religious affiliations (81.8%) compared to those who belong a religion. Most adolescent girls who belong to the Ga/Dangme ethnic group (61.5%) reported having multiple sexual partners compared to adolescent girls with other ethnic groups. The study also found that there were more adolescent girls with multiple sexual partners living in female headed households (58.7%) compared to adolescent girls living in household headed by males (44.4%). Adolescent girls who dwell in household with middle wealth index had a higher proportion (59.8%) engaging in multiple sexual partnerships compared to those who belong to households with rich and poor wealth indexes.

In addition, adolescent girls who indicated consuming alcohol in the past 4 weeks (61.4%) had a higher proportion of them having multiple sexual partners compared to those who never consumed alcohol (44.6%). Multiple sexual partnerships were more prevalent among adolescent girls who used modern contraceptive methods (63.2%) compared to those using traditional methods (51.7%) and no method (43.5%). The experience of multiple sexual partnerships was more prevalent among adolescent girls who had ever tested for HIV (63.9%) compared to those who had never tested for HIV (48.1%). A higher proportion of adolescent girls who indicated having STIs (78.3%) had been engaged in multiple sexual partnerships compared to those who did not have any STIs (49.2%). Adolescent girls who had heard of any STIs (52.6%) and AIDS (52.9%) were more involved in multiple sexual partnerships compared to those who had never heard of these infections.

### Age of first sex and other factors predicting multiple sexual partnerships among adolescent girls 15–19 years in Ghana

Table 4 presents the results of age at first sex and other factors predicting multiple sexual partnerships among adolescent girls aged 15–19 years in Ghana. Model I showed that age at first sex significantly predicted multiple sexual partnerships among adolescent girls. Adolescent girls who had their first sexual intercourse before reaching age 15 were more likely (aOR = 2.00; CI 1.39–2.87) to engage in multiple sexual partnerships compared to those who had their sexual debut before the age of 20.

**Table 4:** Binary logistic regression modelling of age at first sex, individual, household, social and risk factors on multiple sexual partnership among adolescent girls (15–19 years) in Ghana

	Multiple sexual partnership (2 or more)					
Variables	Model I aOR[95% CI]	Model II aOR[95%CI]	Model III aOR [95%CI]	Model IV aOR [95%CI]	Model V aOR [95%CI]	Model VI aOR [95% CI]
Age at first sex						
< 15 years	2.00***[1.39–2.87]	1.85* [1.03–3.31]	1.71 [0.98–2.99]	1.85*[1.02–3.38]	1.71[0.96–3.05]	1.83[0.95–3.53]
≥ 15 to 20 years	**Ref**	**Ref**	Ref	**Ref**	Ref	Ref
Individual level factors						
Educational level						
No Education		0.92[0.19–4.47]				1.63[0.28–9.43]
Primary		0.80[0.36–1.74]				0.68[0.28–1.62]
Secondary +		Ref				Ref
Religion						
Orthodox		7.73[0.36–165.01]				5.72[0.25–133.73]
Pentecostal/charismatic		4.47[0.21–94.57]				3.53[0.15–81.35]
Other christian		7.08[0.33–153.88]				6.21[0.26–147.54]
Islam		2.93[0.12–69.62]				3.68[0.14–95.47]
No Religion		Ref				Ref
Ethnicity						
Akan		0.62[0.21–1.90]				0.82[0.23–2.93]
Ga/Dangme		0.40[0.08–1.91]				0.94[0.16–5.49]
Ewe		0.79[0.23–2.68]				1.14[0.27–4.83]
Mole-Dagbani		0.76[0.22–2.56]				0.93[0.24–3.61]
Gurma		0.60[0.14–2.65]				1.15[0.22–6.04]
Other		Ref				Ref
Community level factors						
Ecological zone of residence						
Northern zone			Ref			Ref
Middle belt			0.89[0.40–2.01]			0.56[0.18–1.73]
Coastal zone			1.22[0.54–2.74]			0.73[0.22–2.42]
Place of residence						
Urban			0.92[0.54–1.55]			0.87[0.44–1.74]
Rural			Ref			Ref
Household level factors						
Sex of household head						
Male				Ref		Ref
Female				2.33**[1.30–4.42]		2.25*[1.18–4.29]
Age of Household Head						
20–29				0.10[0.26–3.82]		1.45[0.34–6.13]
30–39				1.68[0.48–5.83]		2.27[0.60–8.58]
40–49				2.13[0.70–6.55]		2.86[0.85–9.66]
50–59				0.92[0.28–3.07]		1.16[0.32–4.23]
60–69				0.39[0.09–1.81]		0.47[0.10–2.34]
70 +				Ref		Ref
Wealth Index						
Poorest				0.65[0.22–1.87]		0.63[0.16–2.40]
Poorer				0.87[0.34–2.20]		0.96[0.31–2.93]
Middle				0.92[0.38–2.25]		0.91 [0.33–2.52]
Richer				0.41[0.13–1.27]		0.46[0.14–1.51]
Richest				Ref		Ref
Social and Risk level factors						
Consume alcohol in the past 4 weeks						
Did not consume alcohol					1.93*[1.02–3.65]	2.00[1.00–4.04]
Consumed alcohol					2.12*[1.08–4.16]	2.38 *[1.09–5.17]
Never consumed alcohol					Ref	Ref
Contraceptive use						
Use no method					Ref	**Ref**
Use traditional method					1.27[0.58–2.77]	1.37[0.60–3.14]
Use modern method					1.92*[1.06–3.49]	1.87[0.99–3.55]
Cigarettes smoking						
Did not smoke					1.62[0.29–1.31]	1.17[1.20–6.90]
Smoke					Ref	Ref
Ever tested for HIV						
No					Ref	**Ref**
Yes					0.68[0.35–1.40]	0.72[0.35–1.49]
Had any STI						
No					0.71[0.32–1.60]	0.66[0.27–1.61]
Yes					Ref	Ref
Ever heard of STI						
No					Ref	Ref
Yes					5.81[0.26–129.52]	8.35[0.32–218.69]
Ever heard of AIDS						
No					Ref	Ref
Yes					0.52[0.09–3.16]	0.34[0.05–2.44]

Model I: age at first sex only predicting multiple sexual partnerships

Model II: age at first sex and individual factors predicting multiple sexual partnerships; -2 log likelihood: 415.551

Model III: age at first sex and community factors predicting multiple sexual partnerships; -2 Log likelihood: 423.801

Model IV: age at first sex and household factors predicting multiple sexual partnerships; -2 Log likelihood: 394.805

Model V: age at first sex and other risk factors predicting multiple sexual partnerships; -2 Log likelihood: 405.338

Model VI: age at first sex, individual, community, household and risk factors predicting multiple sexual partnerships; -2 Log likelihood: 367.255

Ref = Reference category

Secondary +  = secondary and higher educational level

Source: Computed from 2022 Ghana demographic and health survey (GDHS)

**P < 0.01; ***P < 0.001 [asterisk [bolden] indicate significant predictors of multiple sexual partnership]

In Model II, age at first sex once again significantly predicts multiple sexual partnerships among adolescent girls aged 15–19 years in Ghana, after including individual socio-demographic factors in the model. Adolescent girls who had their sexual debut before the age of 15 had higher odds (aOR = 1.85; C.I 1.03–3.31) of having multiple sexual partners compared to those who had their first sex before reaching age 20 years. In the third model, after the inclusion of community level factors, age at first sex was not significant in predicting sexual partnership among adolescent girls. Model IV showed a statistically significant relationship was established between age at first sex and multiple sexual partnership, after the inclusion of household factors into the model. Adolescent girls who initiated sex before attaining age 15 had higher likelihood (aOR = 1.85; C.I 1.02–3.38) of engaging in multiple sexual partnership compared to those who initiated sex before reaching age 20. The likelihood of having multiple sexual partners is higher among adolescent girls whose household heads are females (aOR = 2.33; C.I 1.30–4.42) than adolescents girls dwelling in households headed by males.

In Model V, age at first sex was not significant in predicting multiple sexual partnership among adolescent girls after the inclusion of social and risk level factors in the model. However, some social and risk factors significantly predict multiple sexual partnership. The high probability of having multiple sexual partners was found among adolescent girls who have did not consume alcohol in the past 4 weeks (aOR = 1.93; C.I 1.02–3.65) and those who consumed alcohol (aOR = 2.12; C.I 1.08–4.16) compared to those who never consumed alcohol. Furthermore, higher odds of engaging in multiple sexual partnerships were recorded among adolescent girls using modern methods of contraception (aOR = 1.92; C.I 1.06–3.49) compared to adolescent girls who are not using any method of contraception.

In the final Model (Model VI), age at first sex did not significantly predict multiple sexual partnership among adolescent girls, after including individual, household, community, social and risk level factors in the model. However, adolescent girls who consumed alcohol in the past 4 weeks (aOR = 2.38; C.I 1.09–5.17) and girls living in female headed households (aOR = 2.25; C.I 1.18–4.29) were more likely to have multiple sexual partners compared to those who never consumed alcohol.

## Discussion

The study examined the effect of age at first sexual intercourse and other determinants (individual, household and risk factors) predicting multiple sexual partnerships among adolescent girls aged 15–19 in Ghana using the 2022 GDHS data. The results show that the average age at first sexual intercourse among adolescent girls in Ghana is 15.5 years. However, earlier studies in Ghana [12, 14, 35] have reported a relatively lower average age at sexual debut among adolescent girls ranging from 13 to 15 years. Findings from other countries in the SSA reported a relatively higher average age at sexual debut among adolescent girls, particularly in Ethiopia (17.6 years) [34], Kenya (16 years) [22], Mali (17.8 years) [46] and Nigeria (15 years) [16]. The variations in average age at first sex and the prevalence of multiple sexual partners between Ghana and other SSA countries can be attributed to cultural, socioeconomic, educational and health related differences. It is worth noting that the age at which adolescent girls initiate sex in Ghana is below the legal consensual age of sex (16 years) and this has implications for their sexual health and well-being in preventing unplanned pregnancies, abortion, HIV/AIDS and other STIs.

Furthermore, the majority (51.6%) of adolescent girls are engaged in multiple lifetime sexual partnerships. Earlier studies in SSA reported a lower prevalence of multiple lifetime sexual partnerships among adolescent girls which ranges from 3.6 to 35.3% [12, 18, 28, 33, 36]. Studies have argued that adolescent girls engage in multiple sexual partnerships for transactional purposes and the exchange of gifts [47, 48]. Thus, adolescent girls have multiple sexual partners for economic and financial reasons [21].

The multilevel regression model indicates that adolescent girls who had their sexual debut before reaching age 15 are more likely to engage in multiple sexual partnerships compared to girls who had their first sexual intercourse before attaining the age of 20 before and after controlling for the effect of other determinants. The high prevalence of multiple sexual partnerships among girls who initiate sex at an earlier age as found by this study, is corroborated by the findings of previous studies in SSA [15–18, 22, 34, 36, 39, 43] and Asia [32]. Reasons associated with high multiple sexual partnerships among adolescent girls who initiate sex at an early age include a lack of maturity and experience to navigate complex relationships and make informed decisions about sexual behaviour. This can lead to a higher likelihood of engaging in risky sexual behaviours, including having multiple partners [42]. Furthermore, adolescent girls who initiate sexual activity early may do so as a way to seek validation of their self-worth; therefore, they believe engaging in sexual relationships with multiple partners can be a misguided attempt to gain acceptance or feel valued by others [48]. Studies also found that adolescent girls who grow up in environments characterised by poverty, violence, substance abuse, or unstable family situations may be more likely to engage in early sexual activity and subsequently have multiple sexual partners [49, 50].

Adolescent girls who belong to households headed by females have higher odds of having multiple sexual partners compared to those residing in households headed by males. Similar findings were reported by an earlier study in South Africa [51], which found a positive association between adolescent girls residing in female-headed homes and having multiple sexual partners. The plausible explanation for this phenomenon is that female-headed households are often associated with lower socioeconomic status, which can lead to inadequate social support systems. Adolescent girls in such households might engage in multiple sexual partnerships as a means of survival and economic support. Additionally, in female-headed households, the primary caregiver might have multiple roles and responsibilities, potentially leading to less oversight of adolescent girls’ activities compared to households headed by males. This reduced supervision could increase the likelihood of engaging in multiple sexual partnerships.

Studies have established strong statistically significant relationship between exposure of adolescent to alcohol consumption and risky sexual behaviour [52–54]. Our findings showed that adolescent girls who consume alcohol and did not consume alcohol in the last 4 weeks preceding the survey were more likely to engage in multiple sexual partnership. This finding corroborates with the findings of previous studies [55, 56] which reported multiple sexual partnership among adolescent and young people who are users of psychoactive substances such as alcohol. Some possible explanations are alcohol consumption affects decision-making and impulse control, making adolescents more likely to engage in risky behaviours such as unprotected sex or having multiple partners without considering the consequences [57]. Again, alcohol lowers inhibitions, making individual more likely to take risks or engage in behaviours they might avoid when sober, including sexual activity with multiple partners [58]. Additionally, drinking of alcoholic beverages is often associated with social settings where adolescents may feel peer pressure to conform to group norms, which may include both drinking and engaging in sexual activities [59]. This can be especially influential in social circles where having multiple sexual partners is normalized or encouraged.

Adolescent girls who reported using modern methods of contraception were more likely to have multiple sexual partners compared to girls who indicated using no method of contraception. Similar findings were reported by earlier studies [22, 30]; other studies found lower contraceptive use among adolescents engaging in multiple sexual partnerships [31, 60]. Reasons for high contraceptive prevalence among adolescent girls with multiple sexual partners include better access to sexual education and reproductive health services, which could increase their knowledge about contraception methods. This increased knowledge might also lead to a greater understanding of safe sex practices, potentially reducing fears of unplanned pregnancies and making them more likely to engage in sexual activity with multiple partners. Similarly, adolescent girls who are using modern contraceptives might perceive themselves as being at a lower risk of negative sexual outcomes due to the efficacy of the method. This perceived lower risk could encourage them to engage in risky sexual behaviour, including multiple sexual partnerships.

### Strengths and limitations of the study

This strength of this study lies in its utilisation of a nationally representative sample of sexually active adolescent girls aged 15–19 years, allowing for the generalisation of results to the entire country and other similar settings. Furthermore, it employs an approach that highlights the contribution of individual demographic, social, household and risk factors influencing sexual behaviours among adolescents. As a result, this study provides valuable information for policymakers to design and implement programmes targeting risk-taking behaviours, such as multiple sexual partnerships among adolescents amidst the identified multiple factors.

However, despite these strengths, the study is subject to some limitations. Firstly, as a cross-sectional study, causality cannot be determined. Additionally, there is a possibility of reporting bias, misreporting underreporting, exaggeration and socially desirable responses regarding sexual activity and the number of sexual partners, given the sensitive nature of the subject under study.

Another limitation is the secondary nature of the data, which did not allow the inclusion and exploration of other predicting factors such as physiological and psychological factors, parental guidance and communication, which could potentially influence the association between age at first sex and the number of sexual partners.

## Conclusion

The study concludes that an increasing proportion of adolescent girls aged 15–19 years are engaging in multiple sexual partnerships in Ghana. Furthermore, it found that girls initiating sex at an earlier age are at an increased risk of having multiple sexual partners. Factors such as residing in female-headed households and households with a middle wealth index, using modern contraceptives, and ever testing for HIV increase the risk of multiple sexual partnerships among adolescent girls in Ghana. Based on these findings, policies promoting delayed sexual debut and empowering adolescents, particularly those from low socio-economic backgrounds, to make informed decisions are recommended for enhancing quality sexual health.

## Data Availability

Datasets used for this study are openly available and can be accessed through https://dhsprogram.com/.

## Abbreviations

AIDS: Acquired Immunodeficiency Syndrome
aOR: Adjusted odds ratio
CI: Confidence interval
GDHS: Ghana demographic and health survey
HIV: Human immunodeficiency virus
PHC: Population and housing census
SSA: Sub-Saharan Africa
SDG: Sustainable development goal
STI: Sexually transmitted infection
WHO: World Health Organisation

## References

[1] Lenz B. The transition from adolescence to young adulthood: a theoretical perspective. J Sch Nurs. 2001;17(6):300–6. 11804406 10.1177/10598405010170060401

[2] Tulloch T, Kaufman M. Adolescent sexuality. Pediatr Rev. 2013;34(1):29–38. 23281360 10.1542/pir.34-1-29

[3] Olesen TB, Jensen KE, Nygård M, Tryggvadottir L, Sparén P, Hansen BT, Liaw KL, Kjær SK. Young age at first intercourse and risk-taking behaviours—a study of nearly 65 000 women in four Nordic countries. Eur J Public Health. 2012;22(2):220–4. 21596800 10.1093/eurpub/ckr055

[4] Falb KL, Annan J, Kpebo D, Cole H, Willie T, Xuan Z, Raj A, Gupta J. Differential impacts of an intimate partner violence prevention program based on child marriage status in rural Côte d’Ivoire. J Adolesc Health. 2015;57(5):553–8. 26372368 10.1016/j.jadohealth.2015.08.001 PMC5783193

[5] World Health Organization. Committing to implementation of the Global Strategy for Women’s, Children’s and Adolescents’ Health (2016–2030): technical report. Geneva: World Health Organization; 2023.

[6] Nations U. Department of economic and social affairs. population division. 2015.

[7] World Health Organization. Child and adolescent health in humanitarian settings: operational guide: a holistic approach for programme managers. Geneva: World Health organization; 2021.

[8] Idele P, Gillespie A, Porth T, Suzuki C, Mahy M, Kasedde S, Luo C. Epidemiology of HIV and AIDS among adolescents: current status, inequities, and data gaps. JAIDS J Acquir Immune Defic Syndr. 2014;1(66):S144–53. 10.1097/QAI.0000000000000176 24918590

[9] UN D. World population prospects 2019: highlights. united nations department for economic and social affairs. 2019

[10] Ferede TA, Muluneh AG, Wagnew A, Walle AD. Prevalence and associated factors of early sexual initiation among youth female in sub-Saharan Africa: a multilevel analysis of recent demographic and health surveys. BMC Womens Health. 2023;23(1):147. 36997947 10.1186/s12905-023-02298-z PMC10061848

[11] Ghana. Statistical Service. Population & housing census of ghana. Ghana Statistical Service: Accra; 2023.

[12] Doku D. Substance use and risky sexual behaviours among sexually experienced Ghanaian youth. BMC Public Health. 2012;12:1–7. 22839700 10.1186/1471-2458-12-571 PMC3517501

[13] Amoako JF. Geographical hotspots and correlates of early sexual debut among women in Ghana. Reprod Health. 2022;19(1):118. 35550601 10.1186/s12978-022-01425-7 PMC9097126

[14] Asante KO, Nketiah-Amponsah E, Andoh-Arthur J, Boafo IM, Ampaw S. Correlates of early sexual debut among sexually active youth in Ghana. Int Q Community Health Educ. 2018;39(1):9–17. 30479192 10.1177/0272684X18811016

[15] Yaya S, Bishwajit G. Age at first sexual intercourse and multiple sexual partnerships among women in Nigeria: a cross-sectional analysis. Front Med. 2018;8(5):171. 10.3389/fmed.2018.00171 PMC6002498 29938205

[16] Alawode OA, Ogunwemimo H, Bolorunduro ME, Awoleye AF. Age at sexual debut and multiple sexual partnerships among adolescents in Nigeria: an assessment of the mediating role of the knowledge of sexually transmitted infections. Adolescents. 2021;1(4):421–32.

[17] Zuma K, Setswe G, Ketye T, Mzolo T, Rehle T, Mbelle N. Age at sexual debut: a determinant of multiple partnership among South African youth. Afr J Reprod Health. 2010;14(2):47–54. 21243918

[18] Osuafor GN, Okoli CE. Factors associated with multiple sexual partners among first-year students in a South African university. Afr J Reprod Health. 2021;25(4):69–78. 37585860 10.29063/ajrh2021/v25i5.7

[19] Harrison A, Cleland J, Frohlich J. Young people’s sexual partnerships in KwaZulu-Natal, South Africa: patterns, contextual influences, and HIV risk. Stud Fam Plann. 2008;39(4):295–308. 19248716 10.1111/j.1728-4465.2008.00176.x PMC3848499

[20] Doyle AM, Plummer ML, Weiss HA, Changalucha J, Watson-Jones D, Hayes RJ, Ross DA. Concurrency and other sexual partnership patterns reported in a survey of young people in rural Northern Tanzania. PLoS ONE. 2017;12(8): e0182567. 28837686 10.1371/journal.pone.0182567 PMC5570426

[21] Ningpuanyeh WC, Sathiya SA. Correlates of early sexual debut and its associated STI/HIV risk factors among sexually active youths in Malawi. J Asian Afr Stud. 2017;52(8):1213–24.

[22] Rositch AF, Cherutich P, Brentlinger P, Kiarie JN, Nduati R, Farquhar C. HIV infection and sexual partnerships and behaviour among adolescent girls in Nairobi, Kenya. Int J STD AIDS. 2012;23(7):468–74. 22843999 10.1258/ijsa.2012.011361 PMC3571685

[23] Stephenson R, Simon C, Finneran C. Community factors shaping early age at first sex among adolescents in Burkina Faso, Ghana, Malawi, and Uganda. J Health Popul Nutr. 2014;32(2):161. 25076654 PMC4216953

[24] Clark S. Early marriage and HIV risks in sub-Saharan Africa. Stud Fam Plann. 2004;35(3):149–60. 15511059 10.1111/j.1728-4465.2004.00019.x

[25] Wellings K, Collumbien M, Slaymaker E, Singh S, Hodges Z, Patel D, Bajos N. Sexual behaviour in context: a global perspective. The Lancet. 2006;368(9548):1706–28. 10.1016/S0140-6736(06)69479-8 17098090

[26] Wight D, Henderson M, Raab G, Abraham C, Buston K, Scott S, Hart G. Extent of regretted sexual intercourse among young teenagers in Scotland: a cross sectional survey. BMJ. 2000;320(7244):1243–4. 10797033 10.1136/bmj.320.7244.1243 PMC27366

[27] Szapocznik J, Coatsworth JD. An ecodevelopmental framework for organizing the influences on drug abuse A developmental model of risk and protection. Washington: American Psychological Association; 1999.

[28] Sathiyasusuman A. Associated risk factors of STIs and multiple sexual relationships among youths in Malawi. PLoS ONE. 2015;10(8): e0134286. 26248328 10.1371/journal.pone.0134286 PMC4527764

[29] Yeboah I, Okyere J, Dey NE, Mensah RO, Agbadi P, Essiaw MN. Multiple sexual partnership among adolescent boys and young men in Ghana: analysis of the 2003–2014 Ghana demographic and health survey. Tropical Med Health. 2022;50(1):88. 10.1186/s41182-022-00484-7 PMC9703711 36443834

[30] Mangold K, Manjengwa PG, Musekiwa A, Kuonza LR. Cognitive and behavioural determinants of multiple sexual partnerships and condom use in South Africa: results of a national survey. Southern Afr J HIV Med. 2019;20(1):1–9. 10.4102/sajhivmed.v20i1.868 PMC6620483 31308963

[31] Exavery A, Lutambi AM, Mubyazi GM, Kweka K, Mbaruku G, Masanja H. Multiple sexual partners and condom use among 10–19 year-olds in four districts in Tanzania: what do we learn? BMC Public Health. 2011;11:1–9. 21696581 10.1186/1471-2458-11-490 PMC3141458

[32] Son DT, Oh J, Heo J, Huy NV, Minh HV, Choi S, Hoat LN. Early sexual initiation and multiple sexual partners among Vietnamese women: analysis from the Multiple Indicator Cluster Survey, 2011. Glob Health Action. 2016;9(1):29575. 26950566 10.3402/gha.v9.29575 PMC4780093

[33] Gbordzoe NI, Obeng P, Ogum MA, Amoadu M, Sarfo JO, Hagan Jnr JE. Multiple sexual partnership among school-going adolescents in Benin: a population-based study of prevalence and predictors. Dis Soc Sci Health. 2023;3(1):23.

[34] Yosef T, Nigussie T, Getachew D, Tesfaye M. Prevalence and factors associated with early sexual initiation among college students in Southwest Ethiopia. Biomed Res Int. 2020;25:2020. 10.1155/2020/8855276 PMC7710405 33299885

[35] Akumiah PO, Suglo JN, Sebire SY. Early life exposures and risky sexual behaviors among adolescents: a cross-sectional study in Ghana. Nigerian Med J. 2020;61(4):189. 10.4103/nmj.NMJ_100_20 PMC7688025 33284892

[36] Muchiri E, Odimegwu C, Banda P, Ntoimo L, Adedini S. Ecological correlates of multiple sexual partnerships among adolescents and young adults in urban Cape Town: a cumulative risk factor approach. Afr J AIDS Res. 2017;16(2):119–28. 28639475 10.2989/16085906.2017.1318762

[37] Omona K, Ssuka JK. Early sexual debut and associated factors among adolescents in Kasawo Sub-county, Mukono district, Uganda. Cogent Public Health. 2023;10(1):2183561.

[38] Adongo BW. Assessing factors influencing early sexual initiation among adolescents (13 to 19 Years) in Ghana: a qualitative study. Int J Caring Sci. 2018

[39] Demographic G. Health survey (2008) Accra. Ghana: GSS, GHS, and ICF Macro. 2009.

[40] Demographic G. Health survey (2022) Accra. Ghana: GSS, GHS, and ICF Macro. 2023.

[41] Fatusi AO, Blum RW. Predictors of early sexual initiation among a nationally representative sample of Nigerian adolescents. BMC Public Health. 2008;8:1–4. 18439236 10.1186/1471-2458-8-136 PMC2390536

[42] Tuoyire DA, Anku PJ, Alidu L, Amo-Adjei J. Timing of first sexual intercourse and number of lifetime sexual partners in sub-Saharan Africa. Sex Cult. 2018;22:651–68.

[43] Sprecher S, O’Sullivan LF, Drouin M, Verette-Lindenbaum J, Willetts MC. The significance of sexual debut in women’s lives. Curr Sex Health Rep. 2019;11:265–73.

[44] Klu D, Kyei-Arthur F, Appiah M, Odame ML. Multilevel predictors of anaemia among pregnant women in Ghana: New evidence from the 2019 Ghana Malaria Indicator Survey. PLOS Global Public Health. 2024;4(9): e0003673. 39236009 10.1371/journal.pgph.0003673 PMC11376585

[45] Fenta SM, Biresaw HB, Fentaw KD. Risk factor of neonatal mortality in Ethiopia: multilevel analysis of 2016 demographic and health survey. Trop Med Health. 2021;49:1–1. 33541435 10.1186/s41182-021-00303-5 PMC7860228

[46] Asare BY, Zegeye B, Ahinkorah BO, Ameyaw EK, Seidu AA, Yaya S. Early sexual debut and its associated factors among young women aged 15–24 in Mali: a multilevel analysis. Arch Sex Behav. 2023;52(6):2491–502. 37069468 10.1007/s10508-023-02591-w

[47] Cropanzano R, Mitchell MS. Social exchange theory: an interdisciplinary review. J Manag. 2005;31(6):874–900.

[48] Cox CM, Babalola S, Kennedy CE, Mbwambo J, Likindikoki S, Kerrigan D. Determinants of concurrent sexual partnerships within stable relationships: a qualitative study in Tanzania. BMJ Open. 2014;4(2): e003680. 24508848 10.1136/bmjopen-2013-003680 PMC3918978

[49] Borne FV. Trying to survive in times of poverty and AIDS: women and multiple partner sex in Malawi. Netherlands: Het Spinhuis Publishers; 2005.

[50] Janighorban M, Boroumandfar Z, Pourkazemi R, Mostafavi F. Barriers to vulnerable adolescent girls’ access to sexual and reproductive health. BMC Public Health. 2022;22(1):2212. 36447192 10.1186/s12889-022-14687-4 PMC9706928

[51] Odimegwu CO, Ugwu NH. A multilevel mixed effect analysis of neighbourhood and individual level determinants of risky sexual behaviour among young people in South Africa. Reprod Health. 2022;19(1):119. 35549967 10.1186/s12978-022-01407-9 PMC9096753

[52] Moore EW, Berkley-Patton JY, Hawes SM. Religiosity, alcohol use, and sex behaviors among college student-athletes. J Relig Health. 2013;52:930–40. 21979810 10.1007/s10943-011-9543-z

[53] Cho HS, Yang Y. Relationship between alcohol consumption and risky sexual behaviors among adolescents and young adults: a meta-analysis. Int J Public Health. 2023;19(68):1605669. 10.3389/ijph.2023.1605669 PMC10154531 37153699

[54] Choudhry V, Agardh A, Stafström M, Östergren PO. Patterns of alcohol consumption and risky sexual behavior: a cross-sectional study among Ugandan university students. BMC Public Health. 2014;14:1–1. 24502331 10.1186/1471-2458-14-128 PMC3933239

[55] Kyei-Arthur F, Kyei-Gyamfi S. Alcohol consumption and risky sexual behaviors among fishers in Elmina in Ghana. BMC Public Health. 2023;23(1):1328. 37434125 10.1186/s12889-023-16239-w PMC10337065

[56] Livingston JA, Bay-Cheng LY, Hequembourg AL, Testa M, Downs JS. Mixed drinks and mixed messages: adolescent girls’ perspectives on alcohol and sexuality. Psychol Women Q. 2013;37(1):38–50. 23833392 10.1177/0361684312464202 PMC3699882

[57] Wagenaar C, Florence M, Adams S, Savahl S. Factors influencing the relationship between alcohol consumption and risky sexual behaviour among young people: a systematic review. Cogent Psychology. 2018;5(1):1483049.

[58] Carels C, Florence M, Adams S, Sinclair DL, Savahl S. Youths’ perceptions of the relation between alcohol consumption and risky sexual behaviour in the Western Cape, South Africa: a qualitative study. Child Indic Res. 2022;15(4):1269–93. 35079296 10.1007/s12187-022-09913-9 PMC8773401

[59] Morris H, Larsen J, Catterall E, Moss AC, Dombrowski SU. Peer pressure and alcohol consumption in adults living in the UK: a systematic qualitative review. BMC Public Health. 2020;20:1–3. 32631278 10.1186/s12889-020-09060-2 PMC7339443

[60] Kalichman SC, Ntseane D, Nthomang K, Segwabe M, Phorano O, Simbayi LC. Recent multiple sexual partners and HIV transmission risks among people living with HIV/AIDS in Botswana. Sexually Transmit Infect. 2007;83(5):371–5. 10.1136/sti.2006.023630 PMC2659030 17475684

